# Antimicrobial susceptibility profiling and genomic diversity of *Acinetobacter baumannii* isolates: A study in western Iran

**Published:** 2013-09

**Authors:** Parviz Mohajeri, Abbas Farahani, Mohammad Mehdi Feizabadi, Hosnieh Ketabi, Ramin Abiri, Farid Najafi

**Affiliations:** 1 Department of Microbiology, School of Medicine, Kermanshah University of Medical Sciences, Kermanshah, Iran; 2 Student Research Committee, Kermanshah University of Medical Sciences, Kermanshah, Iran; 3 Department of Microbiology, School of Medicine, Tehran University of Medical Sciences, Tehran, Iran; 4 Kermanshah Health Research Center (KHRC), School of Health, Kermanshah University of Medical Sciences, Kermanshah, Iran

**Keywords:** Acinetobacter, beta-lactamase, carbapenemase, Pulsed-Field Gel Electrophoresis, Kermanshah

## Abstract

**Background and Objective:**

*Acinetobacter baumannii* is an aerobic non-motile Gram-negative bacterial pathogen that is resistant to most antibiotics. Carbapenems are the most common antibiotics for the treatment of infections caused by this pathogen. Mechanisms of antibiotic-resistance in *A. baumannii* are mainly mediated by efflux pumps-lactamases. The aim of this study was to determine antibiotic susceptibility, the possibility of existence of OXAs genes and fingerprinting by Pulsed-Field Gel Electrophoresis (PFGE) among clinical isolates of *Acinetobacter* collected from Kermanshah hospitals.

**Materials and Methods:**

One hundred and four isolates were collected from patients attending Imam Reza, Taleghani and Imam Khomeini hospitals of Kermanshah (Iran). Isolates were identified by biochemical tests and API 20NE kit. The susceptibility to different antibiotics was assessed with Kirby-Bauer disk diffusion method. PCR was performed for detection of *bla*_OXA-23_, *bla*_OXA-24_, *bla*_OXA-51_ and *bla*_OXA-58_ beta-lactamase genes. Clonal relatedness was estimated by PFGE (with the restriction enzyme *Apa* I) and DNA patterns were analyzed by Gel compare II 6.5 software.

**Results:**

All isolates showed high-level of resistance to imipenem, meropenem as well as to other antimicrobial agents, while no resistance to polymyxin B, colistin, tigecylcine and minocycline was observed. The *bla*_OXA-23like_ and *bla*_OXA-24 like_ were found among 77.9% and 19.2% of the isolates, respectively. All isolates were positive for *bla*_OXA-51_, but none produced any amplicon for *bla*_OXA-58_. PFGE genotype analysis suggested the existence of eight clones among the 104 strains [A (n = 35), B (n = 29), C (n = 19), D (n = 10), E (n = 4), F (n = 3), G (n = 3), H (n = 1)]. Clone A was the dominant clone in hospital settings particularly infection wards so that the isolates in this group, compared to the other clones, showed higher levels of resistance to antibiotics.

**Conclusion:**

The *bla*_OXA-51-like_ and *bla*_OXA-23like_ were the predominant mechanisms of resistance to imipenem in *A. baumannii*. A high prevalence of clone A, B and C in different parts of the healthcare system showed that hospitalized patients should be safeguarded to prevent the spread of these clones. Early recognition of the presence of carbapenem-resistant *A. baumannii* clones is useful for preventing their spread within the hospital environment.

## INTRODUCTION

*Acinetobacter baumannii* is an aerobic non-motile Gram-negative coccobacillus and polymorphic bacterial pathogen that is easily spread from one patient to others, persisting in the environment for many days (1). It is the third most common pathogenic bacteria isolated from hospitalized patients with pneumonia that plays a significant role in nosocomial infections (2–3). Acinetobacter is known to be an important nosocomial pathogen, isolated predominantly in intensive care units (ICUs), and responsible for severe infections. *A. baumannii* are usually multidrug resistant (MDR), showing resistance to the third generation cephalosporins, aminoglycosides and fluoroquinolone (4). Carbapenem resistance among *Acinetobacter* spp. has been increasing during the last decade so that carbapenem resistant *A. baumannii* has become a worldwide problem (5, 6). The most common mechanism of imipenem resistance reportedly involves the association between carbapenem-hydrolyzing-β-lactamases belonging to the metallo-β-lactamases (Ambler class B) and oxacillinases (Ambler class D). *A. baumannii* usually hydrolyze oxacillin more efficiently than benzyl penicillin. (6) Class B carbapenemase including 2 important enzymes IMP and VIM that metallo beta lactamases. (7) Extensive use of antimicrobial chemotherapy, particularly carbapenems, has contributed to the appearance of carbapenem-hydrolyzing class D β-lactamases (CHDLs). These enzymes are frequently identified in *A. baumannii*. Identification of a CHDL-encoding gene was first reported in 1995 (8). Four major subgroups of acquired CHDLs have been identified in *A. baumannii*, including OXA-23, OXA-40, OXA-58, and OXA-143 β-lactamase groups in addition to the naturally-occurring OXA-51 β-lactamase (9). The *bla*_OXA-58 like_ has been identified worldwide, but mostly from France, England, Argentina, Spain, Turkey, Romania, Austria, Greece, Scotland, and Kuwait (10). The significant contribution of these enzymes to carbapenem resistance has been emphasized, particularly when they are accompanied by IS*Aba1* and IS*Aba3* in the naturally occurring plasmid (6). Therefore, the present study was aimed to determine the drug susceptibility patterns of *A. baumannii*. We report an evaluation of CHDL-producing *A. baumannii* isolates collected from three Hospitals of Kermanshah (Iran). The isolates were assessed for the presence of genes *bla*_OXA-23_ _like_, *bla*_OXA-24_ _like_, *bla*_OXA-51_ _like_ and *bla*_OXA-58_ _like_ with PCR. Furthermore, in this study, we report an evaluation of CHDL-producing *A. baumannii* isolates collected from various Hospitals of Kermanshah and the association between their susceptibility and genetic profile as well as PFGE typing for total isolates. Pulsed-Field Gel Electrophoresis (PFGE) was performed to investigate the genetic relation among the isolates and determined wide spread clone (11).

## MATERIALS METHODS

### Bacterial identification and antimicrobial susceptibility testing

A total of 104 *Acinetobacter* spp. were cultured from sputum (n = 69), blood (n = 32) and urine (n = 3) clinical specimens in different hospitals of Kermanshah (Iran) during 2010–11. The strains were identified as *A. baumannii* by conventional biochemical tests and API 20NE kit (version 6.0, bio-Mérieux, Marcy L'Etoile, France) (12). All isolates were tested by the Kirby-Bauer method of disk diffusion according to the CLSI guidelines to check their susceptibilities to amikacine (30 μg), ceftriaxone (30 μg), ciprofloxacin (5 μg), trimethoprim/ sulfamethoxazole (30 μg), gatifloxacine (5 μg), colistin (10 μg), gentamicine (10 μg), imipenem (10 μg), meropenem (10 μg), piperacillin (100 μg), polymyxin B (300 unit), levofloxacin (5 μg), minocycline (30 μg), mezlocilline (75 μg), tetracycline (30 μg), tobramycine (10 μg), cefepime (30 μg), cephpodoxime (10 μg), cefotaxime (30 μg), ceftazidime (30 μg), rifampicine (5 μg) (MAST, Merseyside, UK) (13).

### MBL and ESBL screening

The isolates were identified for a phenotypic MBL screening with the E-test MBL (bio-Mérieux, Marcy L'Etoile, France) as per the manufacturer's instructions (14). Indistinguishable MBL producing isolates were detected and confirmed by Cica-Beta-Test Strip (Kanto Chemical, Tokyo) as per the manufacturer's instructions.

Screening for ESBL-producing organisms was carried out using Double Disk Synergy Test (DDST). Those strains that showed resistance to imipenem by disk diffusion test were re-checked in T-test (15).

### PCR amplification of OXA genes

PCR screening was performed for the carbapenemase-encoding genes (*bla*_OXA-23-like_, *bla*_OXA-24-like_, *bla*_OXA-51-like_ and *bla*_OXA-58-like_). PCR analysis was performed using the primers described by Kuo *et al*. (16).

### Pulsed-field gel electrophoresis (PFGE)

All *A. baumannii* isolates were analyzed by CHEF Mapper PFGE according to the protocols previously described by Durmaz *et al*. with little modifications (17). We used *A. baumannii* ATCC 19606 for normalization of the gel (17, 18). The solution absorption coefficient was set at 600 nm. The cell lysis solution I consisted of Tris-HCl 50 mM (pH 8), EDTA 50 mM (pH 8), 1 mg/ml proteinase K and 1.5 mg/ml lysozyme (Roche Applied Sciences) that was kept shaking in water bath at 37°C for 45 min. Lysis solution II consisted of EDTA 0.5 M (pH 8), 0.4 mg/ml proteinase K and 1% sarcosyl was transferred to tubes containing plugs while kept shaking in water bath at 50°C for 2 h. Digestion of all organisms were performed with *ApaI* (New England Biolabs) restriction enzyme. One half of each plug was transferred to 2 ml tubes that contains 100 μl buffer 1X consisting of *ApaI* enzyme, and then was incubated at 25°C for 2 h.

### Electrophoresis

1% (w/v) of pulsed-field Mega-base Agaros (Bio-Rad laboratories) added into 100 ml of TBE 0.5X (1 liter of 5X stock solution with pH 8). DNA separation was performed in TBE 0.5X (pH 8) buffer in a pulsed-field electrophoresis system (CHEF Mapper; Bio-Rad Laboratories, Hercules, CA, USA) by program two state with the following conditions: temperature 14°C; voltage 6 V/cm; switch angle, 120°; switch ramp 2.2–35 s for 19 h. We used Lambda Ladder PFGE Marker (NEB: N0340) as molecular size marker. The Dice coefficient was used to calculate similarities, and the unweighted paired group method based on average linkages (UPGMA) was used for cluster analysis with Gel compare II version 6.5 (Applied Maths, St Martens-Latem, Belgium) and with a high similar pattern (similarity > 87%) were considered to be derived from a cluster (closely related strains) (17–19).

### Statistical analysis

Data were recorded and entered into a database. Statistical analyses were performed using Stata (Version 11.0). Continuous variables were compared using one-way analysis of variance. Variables were analyzed by chi-square test. A P-value of <0.05 was set as the level of statistical significance for all analyses in the study.

## RESULTS

A total of 104 clinical isolates of *A. baumannii* were collected from 3 hospitals in Kermanshah (Iran). The results showed high-level of resistance to imipenem (79.8%) and meropenem (75%) as well as resistance to other antibiotics is shown in (Table 1). Resistance was observed against polymyxin B (13.5%), minocycline (16.3%), colistin (10.6%). Resistance rates for tigecylcine was low resistance (2.9%). 84 isolates (80.8%) produced MBLs. In our study, 85.6% (n = 89) of isolates were able to produce carbapenemase that was significantly associated with imipenem and meropenem resistance (p value <0.001). Furthermore, 32.7% (n = 34) of isolates showed multidrug resistance phenotype, (MDR, resistant to third generation cephalosporins, amino glycosides and fluoroquinolone) and 8.7% (n = 9) pandrug resistant (PDR, resistant to all available antibacterial agents except polymyxin B and colistin) phenotype, whereas none of the isolates was extensively drug resistant phenotype (XDR). XDR has been defined for the strains showing resistance to some of the most effective anti-bacterial drugs (20). Using PCR assay 77.9% and 19.2% of the isolates were positive for *bla*_OXA-23like_ and *bla*_OXA-24_ _like_ genes, respectively. While all isolates were positive for *bla*_OXA-51like_, none gave any amplicon for the *bla*_OXA-58_ _like_. Co-existence of *bla*_OXA-23_ and *bla*_OXA-24_ like was observed among 16.4% of the isolates. The co-relations between carbapenemase and *bla*_OXA-23_ _like_ was statistically significant (P value 0.002).

**Fig. 1:**
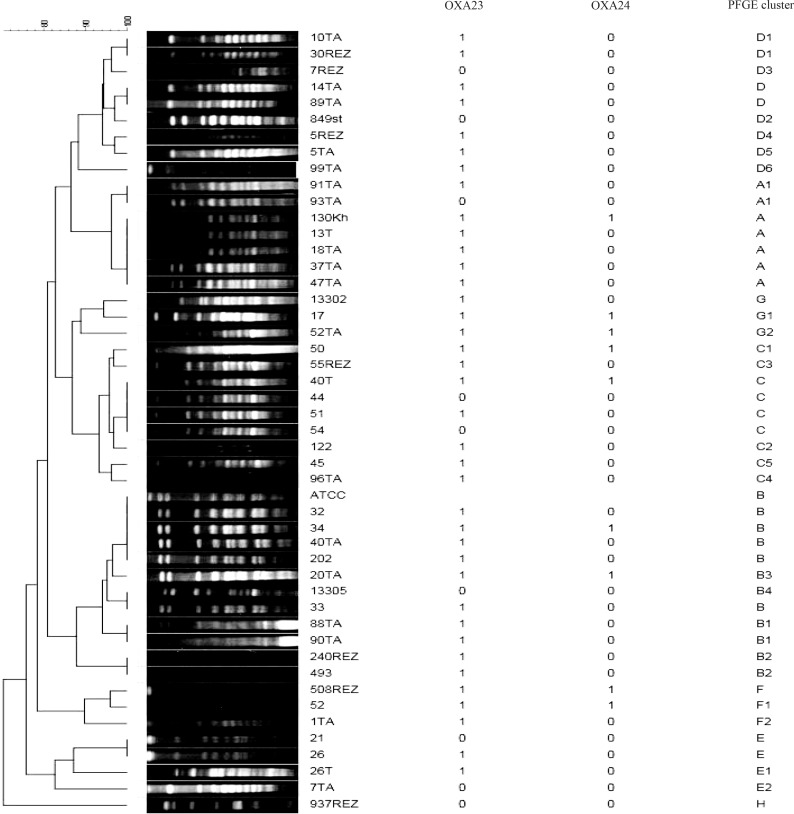
Pulsed-field gel electrophoresis (PFGE) dendrogram and polymerase chain reaction (PCR) of *Acinetobacter baumannii*. Isolates (1= positive, 0= negative for gene)

According to Tenover's criteria(18), a major PFGE type (clone A) containing 35 isolates was identified in 3 studied hospitals in this study. A subtyped designated as A1 was found for type A that differed in migration of one to three bands (Fig. 1). Other PFGE patterns include types B (n = 29), C (n = 19), D (n = 10), E (n = 4), F (n = 3), G (n = 3) and H (n = 1)]. The types A, B, C and D were the dominant types found in the hospitals. Type H consisted of single isolate. The PFGE analysis revealed that 18 isolates were closely related. All iso-lates from infection wards belong to clone A (Table 2). All isolates within type F (100%) showed high-level of resistance to imipenem followed by type A (91.4%) (P value 0.03). In addition, 97.1% of the isolates with type A (n = 89) were able to produce carbapenemase (P value 0.023) (Table 2).

**Fig. 2:**
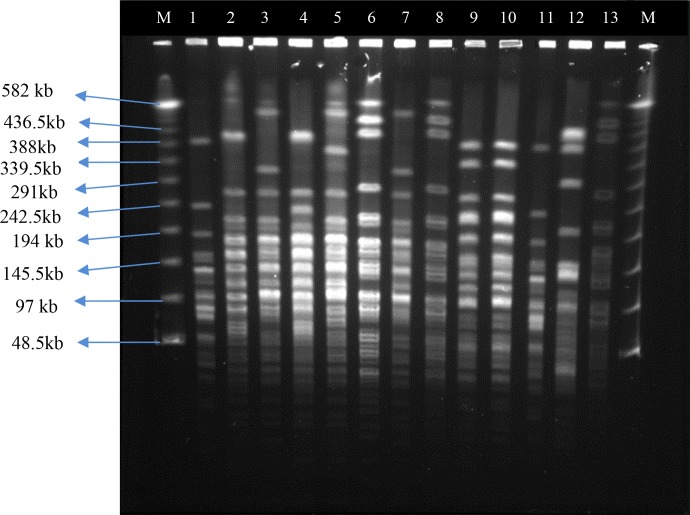
CHEF Profiles of *A. baumannii* strains isolated from different patients. Lateral lanes contain Lambda Ladder PFG Marker, (8 and 13) *A. baumannii* ATCC 19606. (1, 2 and 4) clone D, D3 and D1, (9 and 10) clone A, (3, 7) clone C5 and C, (5) G1, (6) B, (11) clone F, (12) clone H.

## DISCUSSION

Carbapenem resistance has been increasingly common issue among *A. baumannii* isolates in Iranian hospitals in recent years. This study was aimed to evaluate the molecular epidemiology of carbapenem-resistant *A. baumannii* in Kermanshah hospitals, with the aim of identifying predominant clonal circulating. Our study showed low susceptibility rates to most of the clinically available antimicrobial agents for the treatment of *A*. baumannii-induced infections. There was a resistance to polymyxin B, colistin, minocycline, whereas low resistance to tigecylcine (Table 1). This antibiotic can be helpful in treating *A. baumannii*-related infections in hospital settings. This study reports one of the first large outbreaks of MDR 32.7% *A. baumannii* in west of Iran. The global incidence of meropenem resistance in *A. baumannii* was approximately 6% in 1998 but it has dramatically increased to approximately 29% in 2005 (10). This rapid increase has also been happened in the Kermanshah hospitals, where resistance rates to imipenem and meropenem were 79.8% and 75%, respectively. Findings of the present study showed that susceptibility to tigecylcine in 82 out of 83 (98.7%) isolates was significant. A similar increase in the resistance of *A. baumannii* isolates to imipenem and meropenem was found in the previous studies conducted in Iran during 2006–2010 (5–21). In addition, *A. baumannii* isolates (obtained in 2006–2007) from Singaporean hospitals were also highly resistant to carbapenems (22). All the clinical *A. baumannii* isolates obtained in 2006–2007 from Malaysia, exhibited high resistance to all the examined antimicrobial agents except for polymyxin B (Different mechanisms are involved in the *A*. *baumannii* resistance to imipenem. β-Lactamase is important factor to carbapenem-resistance (23). The acquisition of carbapenem resistance in *A. baumannii* is mainly because of the production of two types of β-lactamases: Metallo- β-lactamases (MBLs) and carbapenem-hydrolysing class D β-lactamases (CHDLs). The CHDLs such as *bla*_OXA-23_, *bla*_OXA-24_, *bla*_OXA-51_ appear to be more prevalent and important to carbapenem resistance in this bacterium in some countries including Bahrain, UAE and Kuwait (10). However, the *bla*_OXA-23-like_ and *bla*_OXA-24-like_ genes were responsible for the majority of carbapenem resistance in the isolates studied in this research.

**Table 1:** Antimicrobial-susceptibility for *Acinetobacter baumannii* isolates.

Antimicrobial	Susceptibility; no. (%) of isolates:		

	Susceptible	Intermediate	Resistant
Amikacine	37 (36.6)	10 (9.6)	56 (53)
Ceftriaxone	3 (2.9)	6 (5.8)	95 (91.3)
Ciprofloxacin	32 (30.8)	0(0)	72 (69.2)
Sulfamethoxazole	45 (43.3)	1 (1)	58 (55.8)
Gatifloxacine	51 (49)	8 (7.7)	45 (43.3)
Colistin	93 (89.4)	0 (0)	11 (10.6)
Gentamicine	31 (29.8)	2 (1.9)	71 (68.3)
Imipenem	17 (16.3)	4 (3.8)	83 (79.8)
Meropenem	20 (19.2)	6 (5.8)	78 (75)
Piperacillin	21 (20.2)	3 (2.9)	80 (76.9)
Polymyxin B	90 (86.5)	0 (0)	14 (13.5)
Ceftazidime	30 (28.8)	1 (0.96)	73 (70.2)
Levofloxacin	33 (31.7)	6 (5.8)	65 (62.5)
Minocycline	79 (76)	8 (7.7)	17 (16.3)
Mezlocilline	15 (14.4)	4 (3.8)	85 (81.7)
Tetracycline	30 (28.8)	1 (0.96)	73 (70.2)
Tobramycine	57 (54.8)	6 (5.8)	41 (39.4)
Tigecylcine	100 (96.2)	1 (0.96)	3 (2.9)
Cefepime	26 (25)	2 (1.9)	76 (73.1)
Cephpodoxime	3 (2.9)	0 (0)	100 (96.2)
Cefotaxime	5 (4.8)	1 (0.96)	97 (93.3)
Rifampicine	11 (10.6)	6 (5.8)	87 (83.7)
AMP-Sulbactam	66 (63.5)	3 (2.9)	35 (33.7)

**Table 2:** Comparison of PFGE pattern with antimicrobial susceptibility, OXA genes and source (wards) of isolates (%).

CloneAntibiotics, genes and wards	Clone A	Clone B	Clone C	Clone D	Clone E	Clone F	Clone G	Clone H	*P-value*
IPM	32(91)	20(69)	17(89)	7(70)	3(75)	3(100)	1(33)	0	0.030
MEM	31(88)	19(65)	13(68)	8(80)	2(50)	3(100)	2(66)	0	0.146
CRO	32(91)	27(93)	18(94)	10(100)	3(75)	3(100)	1(33)	1(100)	0.027
CPD	35(100)	29(100)	19(100)	9(90)	2(50)	3(100)	2(66)	1(100)	<0.001
AMP	35(100)	29(100)	18(94)	9(90)	2(50)	3(100)	3(100)	1(100)	<0.001
SXT	20(57)	15(51)	14(73)	2(20)	3(75)	1(33)	3(100)	0	0.079
TGC	1(2)	0	0	2(20)	0	0	0	0	0.094
Carbapenemase	34(98)	22(76)	17(89)	7(70)	4(100)	3(100)	2(66)	0	0.02
MBL	30(85)	22(75)	16(84)	7(70)	3(75)	3(100)	2(66)	1(100)	0.854
ESBL	19(54)	12(42)	12(63)	5(50)	2(50)	1(33)	1(33)	0	0.768
MDR	12(34)	10(34)	7(36)	1(10)	1(25)	2(66)	1(33)	0	0.705
PDR	4(11)	2(7)	2(10)	1(10)	0	0	0	0	0.823
Oxa23	28(80)	23(79)	16(84)	7(70)	1(25)	3(100)	3(100)	0	0.081
Oxa24	7(20)	5(17)	3(15)	0	1(25)	2(66)	2(66)	0	0.113
Urgency	6(17)	2(7)	3(15)	1(10)	1(25)	1(33)	1(33)	1(100)	0.198
ICU	21(60)	24(82)	15(79)	8(80)	3(75)	2(66)	2(66)	0	0.198
Children	1(2.8)	3(10)	1(5.2)	1(10)	0	0	0	0	0.198
Infection	7(20)	0	0	0	0	0	0	0	0.198

IPM: Imipenem, MEM: Meropenem, CRO: Ceftriaxone, CPD: Cephpodoxime, AMP: Ampicillin, SXT: trimethoprim/sulfamethoxazole, TGC: Tigecylcine, MBL:, ESBL: extended-spectrum-beta-lactamase, MDR: multidrug resistant, PDR: pandrug resistant, ICU: intensive care units, P marked in bold if <0.05.

Carbapenemase were found in 89 strains (85.6%), including 74 (83.2%) *bla*_OXA-23like_ that the *bla*_OXA-23like_ was associated with carbapenemase (P = 0.002) because the *bla*_OXA-23like_ gene is associated with Tn2006 that is capable of transposing from bacteria to bacteria could be located on the plasmid or a chromosome (24). *bla*_OXA-23like_ was associated with imipenem (P 0.002) and also with meropenem (P < 0.001). In conclusion, *bla*_OXA-51-like_ and *bla*_OXA-23like_ were the predominant mechanisms of resistance to imipenem in *A. baumannii*. However, for a global epidemiologic analysis, further studies in large scale and different places should be conducted.

Various molecular typing systems have been developed so far to facilitate better understanding of epidemiology of infection with *A. baumannii* (11). This is the first published study of PFGE typing among the clinical strains of *A. baumannii* conducted in west of Iran. We obtained 8 clones and 20 sub clone. Clone A was involved in the majority of outbreaks in Kermanshah. It occurred at different hospitals wards and was the predominant pattern 100% cultured from Infectious Disease wards. Clone B was the second most common pattern involved in outbreaks. Isolates within this clone were mainly positive for *bla*_OXA-23like_ where clone A was dominant for the presence of this gene. The clone (A, A1) were associated with cefotaxime (P value 0.004) indicating a close relationship with spreading dominant clone. Most of MDR and PDR phenotypes were presented in the clones A, B and C. Clonal outbreaks of *A. baumannii-*induced infections containing different OXA-type carbapenemases were reported in Brazil, Taiwan, Iran, Spain, Malaysia, Italy, Turkey, Korea and Argentina (5, 16, 20, 21–25–26). It is conjectured that these genes are closely associated with outbreaks in some countries. Similarly, the results of this study support the viewpoint indicating clonal spread as the main reason for the increasing trend of imipenem, meropenem resistance as well as other antibiotics studied in the hospitals of this study. It is possible that the transfer of these clones to other wards by staff or hospital equipments. A high prevalence of the clone A, B and C in different parts of the healthcare system showed that hospitalized patients should be highly careful to prevent the spread of these clones. Finally, all isolates collected from the hospital 3 in Kermanshah contained *bla*_OXA-23like_, and all isolates belonged to the clone A; therefore, this clone was responsible for the outbreak in this hospital. Early recognition of the presence of carbapenem-resistant *A. baumannii* clones is useful for preventing their spread within the hospital environment.
